# List of Cases Treated in the Surgical Wards of the Pennsylvania Hospital, and Discharged between the 18th and 31st of October, 1838

**Published:** 1838-11-07

**Authors:** Henry H. Smith

**Affiliations:** Resident Surgeon


### CLINICAL REPORTS.

### PENNSYLVANIA HOSPITAL.

*List of Cases treated in the Surgical Wards of the Pennsylvania Hospital, and discharged between the 18th and 31st of October,* 1838.

[Reported by Henry H. Smith, M.D., Resident Surgeon.]

A case of fracture of the upper third of the femur, near the great trochanter, in a man twenty-two years of age, who was admitted July 26th, 1838. The limb was dressed with Physick's modification of Desault's splints, for the first few weeks, but owing to an attack of mania a potu, and a slough on the heel from the extending band, Dr. N. R. Smith's apparatus was applied for ten days, when the first dressing was resumed. On the one hundred and fourteenth day after admission, the patient was allowed to walk, with pasteboard splints bandaged tight to the limb, and on the 23d October, ten days afterwards, he was discharged, cured—the limb, owing to the circumstances before mentioned, being shortened about three-quarters of an inch.

Another case of fracture of the lower third of the femur, in a boy aged fifteen years, caused by a fall, was admitted August 28th: dressed with Desault's splints, by which constant extension was kept up until September 26th, twenty-nine days after admission, when pasteboard splints were applied. On the 10th October he was permitted to walk about, and on the 23d of the same month, fifty-six days after admission, he was discharged, cured, the limb being, by close measurement, only one-eighth of an inch shorter than the sound one, and causing no limp in his gait.

A third case of fracture of the femur, near the middle, in a woman aged eighty years, caused by a fall on the floor, was admitted August 5th. As there was but little hope of bony union, the limb was only laid on the double inclined plane, and supported by pillows. Gradual prostration ensued, and death took place on the 28th of October, eighty-four days after the accident. A post mortem examination of the limb showed a very great effusion of cartilaginous matter around the fracture, sufficient to have rendered the leg partially useful; but no callus was found. There was no fracture of the cervix femoris, but the crista ilii of the same side had been fractured, and united by the same kind of substance as the femur.

A case of fracture of both bones of the leg, caused by a hogshead rolling against it, was admitted August 28th, and dressed with the fracture box for forty-three days. Pasteboard splints were applied for eight days, and the case was discharged, October 23d, cured, sixty-three days after admission. Owing to the great size of this patient, it was necessary to keep him in bed longer than usual, and also to keep the splints on some time after he commenced walking about.

A case of fracture of the fibula, in a man who had been discharged from the institution, two months previously, cured of a fracture of the same bone, was admitted September 20th: dressed with the fracture box for four weeks, afterwards with splints, and discharged, cured, October 27th, thirty-seven days after admission.

Another case of fracture of the fibula, caused by the falling of a bank of earth, was admitted September 8th, dressed in the usual way, splinted twenty-two days after admission, and discharged, cured, October 31st, forty-nine days after the accident.

A case of fracture of the olecranon, in a man, was admitted September 13th. At the time of admission, the elbow was much inflamed and swollen, the arm was therefore bandaged loosely on *an angular* splint; leeches and lead water applied to it, and the man kept in bed with this dressing for four days. After the subsidence of the inflammation, a straight splint, extending from the shoulder to the ends of the fingers was placed in front of the arm, motion being made in the elbow joint, at each dressing after the first eight or ten days; discharged with but little stiffness of the joint, October 23d, forty days after the accident.

A case of fracture of the humerus, high up, which had been mistaken for a dislocation of the shoulder, and after the employment of extension, sent to the house, was admitted October 5th. The shoulder was much inflamed and swollen. Perfect rest with lead water was ordered for the first twenty-four hours, the arm being only supported in a sling. Afterwards it was dressed with an angular splint from the shoulder to the fingers, on the inside, and three short straight splints extending only from the elbow upwards, on the other sides of the humerus. Discharged, cured, October 27th, twenty-two days after admission. This patient was a boy of twelve years of age, of a good constitution, and had the splints removed five days before his discharge, making only seventeen days of actual treatment.

A case of fracture of the humerus, just above the condyles, in a boy aged ten years, caused by a blow from a brickbat, was admitted October 4th. An angular splint was applied to the front of the arm, and continued until a few days previous to his discharge, which took place October 31st, twenty-seven days after the accident. Some slight stiffness remained at the elbow joint.

A case of fracture of the lower end of the radius was admitted September 24th, and dressed with two straight splints, stuffed well on the side next to the arm, and extending from beyond the fingers to the elbow. No bandage was applied around the arm, previous to the splints being fixed; the padding of the splint preventing any deformity from the pressure of the radius against the ulna. Discharged, cured, October 27th, thirty-three days after the accident. This is the usual mode of dressing this accident practised in the house, and out of a large number treated during the year, scarcely a single one is discharged with deformity.

A case of dislocation of the head of the humerus under the pectoral muscle; reduced in the usual way immediately after entrance. Discharged October 31st, nine days after the accident.

The cases of fracture of the femur included in this report, make seven cases of this accident, which have been admitted within the last six months, and are interesting, inasmuch as they offer specimens of fracture in three different places, and at three different periods of life: youth, adolescence, and old age; for the first patient, although only twenty-two years old, was well developed, and had the appearance of being several years older.

The case, too, of the female of eighty, offers the unusual instance of fracture of the middle of the bone instead of the neck, which is generally the result of a fall at this time of life, especially when the height fallen from is so short.

